# Understanding antibiotic misuse in Kazakhstan: insights from the WHO behavioral survey on COVID-19

**DOI:** 10.1186/s12879-025-11764-y

**Published:** 2025-10-16

**Authors:** Serzhan Nazarbek, Nurzhan Aidossov, Ane Tynyshbayeva, Gulnur Zhakhina

**Affiliations:** 1 Club of Experts under the Senate of the Republic of Kazakhstan, Astana, Kazakhstan; 2 Kazakhstan Association of Healthcare Managers, Astana, Kazakhstan; 3 Institute of Management, The Academy of Public Administration under the President of the Republic of Kazakhstan, Astana, Kazakhstan; 4 Academy of Public Administration under the President of the Republic of Kazakhstan, Astana, Kazakhstan; 5 Department of Medicine, School of Medicine, Nazarbayev University, Astana, Kazakhstan

**Keywords:** Non-prescription antibiotic use, Antimicrobial resistance (AMR), COVID-19 pandemic, Healthcare access disparities, Public health behavior

## Abstract

**Background:**

Antimicrobial resistance (AMR) poses a critical global health challenge, exacerbated by the misuse of antibiotics. The COVID-19 pandemic has heightened this issue, particularly in low- and middle-income countries, where self-medication with antibiotics is common. This study aimed to explore the prevalence, socio-demographic factors, and behavioral drivers of non-prescription antibiotic use in Kazakhstan during the COVID-19 pandemic.

**Methods:**

A cross-sectional online survey was conducted among adults in Kazakhstan using a snowball sampling technique. The questionnaire, adapted from the WHO Europe behavioral insights tool, assessed socio-demographics, COVID-19 perceptions, preventive behaviors, trust in information sources, and beliefs in conspiracy theories.

**Results:**

The study included participants aged 18–74 years, with non-prescription antibiotic use more prevalent among rural residents and caregivers of children under 18 years (*p* < 0.001). Individuals perceiving a high probability and severity of COVID-19 were more likely to self-medicate with antibiotics (*p* < 0.001). Interestingly, low trust in healthcare professionals was significantly associated with antibiotic misuse (*p* < 0.001). Despite widespread adherence to preventive measures, individuals who self-medicated exhibited higher compliance rates with handwashing, mask-wearing, and surface disinfection (*p* < 0.05). Additionally, participants who expressed greater worry about COVID-19-related issues, including losing loved ones or economic instability, were less likely to misuse antibiotics. Belief in conspiracy theories was relatively uncommon but associated with reduced antibiotic misuse in certain scenarios.

**Conclusions:**

The findings underscore the need for targeted public health strategies to address disparities in healthcare access, build trust in medical professionals, and enhance antibiotic stewardship efforts to mitigate AMR risks in Kazakhstan.

**Supplementary Information:**

The online version contains supplementary material available at 10.1186/s12879-025-11764-y.

## Introduction

Antimicrobial resistance (AMR) is a growing global public health threat, driven in part by the misuse and overuse of antibiotics [1, 2]. In 2019, AMR was directly responsible for an estimated 1.27 million deaths worldwide and contributed to an additional 4.95 million death cases [3]. Moreover, according to the review from Lord Jim O’Neill and his team in 2014, 700,000 people die worldwide from drug-resistant infections and this number is projected to escalate till 10 million by 2050 [4]. This burden is disproportionately high in low- and middle-income countries, where poverty and socioeconomic inequalities exacerbate the drivers and consequences of AMR [5].

The economic impact of AMR is equally concerning. Projections from the World Bank estimate that AMR could incur an additional US$ 1 trillion in healthcare costs by 2050, alongside annual global gross domestic product (GDP) losses ranging between US$ 1 trillion and US$ 3.4 trillion by 2030 [6]. These statistics underscore the critical threat AMR poses to the sustainability of healthcare systems worldwide.

Kazakhstan, like many other countries, faces challenges in controlling antibiotic consumption, particularly the widespread use of antibiotics without a prescription. Studies have shown that self-medication with antibiotics is a key driver of AMR [5], yet little is known about the extent of this issue in Kazakhstan, particularly in the context of the COVID-19 pandemic. According to the National Research Center for Health Development, in 2021, 70.5% of expenditure on medicines came directly from the population [7]. Notably, while the share of out-of-pocket spending on medicines had been declining in previous years, it surged by 5% points in 2020, likely reflecting the impact of the COVID-19 pandemic, when many individuals purchased medications independently [7]. Moreover, injudicious antibiotic use is one of the possible drivers of multi-drug resistance [8]. This trend highlights the urgent need for improved regulation and stewardship of antibiotic use to mitigate the dual burden of AMR and financial strain on the public.

This study aims to assess the prevalence and determinants of non-prescription antibiotic use in Kazakhstan during the COVID-19 pandemic, examining socio-demographic factors, trust in healthcare providers, conspiracy beliefs, and adherence to preventive measures. By identifying key drivers of self-medication, our findings can inform public health policies and antimicrobial stewardship programs, contributing to Kazakhstan’s efforts to achieve Sustainable Development Goal (SDG) targets on responsible antibiotic use and improved healthcare access.

## Materials and methods

### Study design and population

A cross-sectional study was conducted using an anonymous online survey to achieve the study objectives from June to September 2020. Social media platforms, including Instagram, WhatsApp, and Telegram, were utilized to distribute the survey link to the general population. Additionally, participants were encouraged to share the questionnaire link with others who might be eligible, employing a snowball sampling technique. This approach aimed to collect responses from a broad sample of the general population, thereby enhancing external validity. In addition, computer assisted telephone interviews were used to reach people without Internet access or other underrepresented populations.

The inclusion criteria for the study were as follows: participants had to be citizens of Kazakhstan, aged 18 or older, residing in the country at the time of the survey, and able to complete the questionnaire in Kazakh, Russian, or English. Ethical approval for the study was obtained from the Local Committee on Bioethics of the National Center for Public Health of the Republic of Kazakhstan (№ 2 dated 11 May 2020). Participants were provided with an informed consent form and information regarding voluntary participation before proceeding to the survey questions. The informed consent to participate was obtained from all the participants of this study.

### Instruments

The questionnaire utilized in this study originates from the COVID-19 Snapshot Monitoring (COSMO) survey, developed by the WHO in partnership with the University of Erfurt, Germany, in March 2020 [9]. The survey is originally in English, but it was translated into Russian and Kazakh by a professional interpreter with expertise in WHO terminology and COVID-19 behavioral science. The translator possesses advanced proficiency in English and is fluent in Russian. Detailed instructions were provided to ensure that the translation prioritized conceptual accuracy over literal translation and employed language that was natural and easily understood by a wide audience. The questionnaire used in this study is attached as a Supplementary file.

The questionnaire was also translated into Kazakh by a professional translator fluent in English, Russian, and Kazakh. Additional language adaptations were made to ensure clarity, simplicity, and conciseness. This process considered the respondents’ comprehension of the questions, aimed to avoid jargon, and took into account factors such as gender and age relevance. The questions were further tailored to the local context of Kazakhstan, reflecting the current epidemiological status of the pandemic and the specific public health measures implemented within the country.

### Questionnaire construct

The questionnaire initially encompassed a wide range of variables, detailed elsewhere [9]. For this study, the analysis focused on specific constructs, including socio-demographics, perception of the probability and severity of COVID-19, readiness and self-perceived capabilities, preventive behaviors, knowledge and self-assessment of compliance with prevention measures, trust in information sources, beliefs in conspiracy theories, and levels of worry.

The socio-demographics section included six questions. The “perception of probability and severity of COVID-19” and “readiness and self-perceived capabilities” sections each contained two questions. The “preventive behaviors” section included seven questions, “knowledge and self-assessment” comprised a single question, “trust in information sources” featured eight questions, “beliefs in conspiracy theories” contained five questions, and “worry” included 14 questions. The full questionnaire is provided in Appendix 1. All questions in these sections utilized a five-point Likert scale (e.g., “Strongly disagree” to “Strongly agree”).

### Statistical analysis

Descriptive statistics were calculated using frequencies and percentages for categorical variables and means with standard deviations for continuous variables. Chi-square tests and Fisher’s exact tests were used to identify significant categorical variables, while Student’s t-tests were employed foe continuous ones. For the analysis, responses on the five-point Likert scale were grouped into two categories. For example, responses to the question “How likely do you think you will become infected with COVID-19?” were categorized as “unlikely” for answers such as “very unlikely,” “unlikely,” and “neutral,” while “likely” and “very likely” were categorized as “probably.” The significance level was set at 0.05, and all analyses were conducted using STATA software version 16.0.

## Results

The socio-demographic characteristics of participants and their perceptions related to COVID-19 are summarized in Table 1. Participants’ ages ranged from 18 to 74 years. The mean age of individuals who used antibiotics without a prescription was 41 years, compared to 39 years for those who did not use antibiotics, with this difference being statistically significant (*p* < 0.001). Participants residing in rural areas and those with children under 18 years of age were more likely to use non-prescription antibiotics (*p* < 0.001 and *p* = 0.006, respectively).

Conversely, no statistically significant associations were observed between antibiotic use and gender, employment as a healthcare worker, or having family members over 60 years of age. However, individuals who perceived a high probability of contracting COVID-19 and believed the infection could be severe were more likely to use antibiotics without a prescription (*p* < 0.001).

**Table 1:** Socio-demographic characteristics of participants and their perceptions related to COVID-19 (*n* = 1,990)

Characteristics	Total	Used antibiotics (*n* = 617; 31%)	Didn’t use antibiotics (*n* = 1,373; 69%)	*p*-value
Age, mean (± SD)	39 (±14)	41 (±15)	39 (±14)	< 0.001
Gender, n (%)				
Female	1,152 (58)	356 (58)	796 (58)	0.908
Male	838 (42)	261 (42)	577 (42)	
Living area, n (%)				
Rural	827 (42)	313 (51)	514 (37)	< 0.001
Urban	1,163 (58)	304 (49)	859 (63)	
Medical worker				
Yes	128 (6)	39 (6)	89 (6)	0.892
No	1,862 (94)	578 (94)	1,284 (94)	
Members younger 18				
Yes	1,303 (65)	429 (70)	874 (64)	0.006
No	569 (29)	151 (24)	418 (30)	
Missing	118 (6)	37 (6)	81 (6)	
Members older 60				
Yes	656 (33)	198 (32)	458 (33)	0.573
No	1,218 (61)	383 (62)	835 (61)	
Missing	116 (6)	36 (6)	80 (6)	
Probability and severity of COVID-19 (perception)				
How likely do you think you will become infected with COVID-19?				
Unlikely	1,073 (54)	292 (47)	781 (57)	< 0.001
Probably	917 (46)	325 (53)	592 (43)	
How severe could a COVID-19 infection be for you?				
Mild	1,094 (55)	283 (46)	811 (59)	< 0.001
Severe	896 (45)	334 (54)	562 (41)	
Readiness and perception of own capabilities				
I know how to protect myself from coronavirus				
Don’t know	709 (36)	216 (35)	493 (36)	0.699
Fully informed	1,281 (64)	401 (65)	880 (64)	
For me, avoiding contracting COVID-19 in the current situation is …				
Difficult	1,260 (63)	358 (58)	902 (66)	0.001
Easy	730 (37)	259 (42)	471 (34)	

Regarding beliefs in conspiracy theories, a relatively small percentage of participants subscribed to such beliefs. Notably, in four out of five related questions, participants who believed that the public is not informed about the “hidden motives” behind global events were less likely to use antibiotics without a prescription, and this association was statistically significant (Table 2).

The survey also revealed that over 60% of respondents expressed low trust in information sources such as newspapers, rumors, social networks, radio stations, and public figures on social media. Among those who used antibiotics without a prescription, 62% reported low trust in medical professionals, a statistically significant finding (*p* < 0.001).

**Table 2:** Believe in conspiracy theories and trust in information sources

Characteristics	Total	Used antibiotics (*n* = 617; 31%)	Didn’t use antibiotics (*n* = 1,373; 69%)	*p*-value
Conspiracy Theories (Perception)				
There are many very important things happening in the world that the public is never informed about				
Lie	1,099 (55)	375 (61)	724 (53)	0.001
Truth	891 (45)	242 (39)	649 (47)	
Politicians usually don’t tell us the true reasons behind their decisions				
Lie	1,090 (55)	384 (62)	706 (51)	< 0.001
Truth	900 (45)	233 (38)	667 (49)	
Government agencies closely monitor all citizens				
Lie	1,280 (64)	414 (67)	866 (63)	0.083
Truth	710 (36)	203 (33)	507 (37)	
Events that seem unrelated are often the result of covert activity				
Lie	1,255 (63)	411 (67)	844 (61)	0.028
Truth	735 (37)	206 (33)	529 (39)	
There are secret organizations that have a very strong influence on political decisions				
Lie	1,254 (63)	416 (67)	838 (61)	0.006
Truth	736 (37)	201 (33)	535 (39)	
Trust in information sources				
I trust television				
Low	1,056 (53)	337 (55)	719 (52)	0.352
High	934 (47)	280 (45)	654 (48)	
I trust daily or weekly newspapers				
Low	1,277 (64)	386 (63)	891 (65)	0.315
High	713 (36)	231 (37)	482 (35)	
I trust conversations with family, friends, colleagues				
Low	1,340 (67)	411 (67)	929 (68)	0.644
High	650 (33)	206 (33)	444 (32)	
I trust consultations with medical professionals				
Low	1,078 (54)	382 (62)	696 (51)	< 0.001
High	912 (46)	235 (38)	677 (49)	
I trust social networks				
Low	1,376 (69)	419 (68)	957 (70)	0.423
High	614 (31)	198 (32)	416 (30)	
I trust the radio station				
Low	1,445 (73)	444 (72)	1,001 (73)	0.662
High	545 (27)	173 (28)	372 (27)	
I rely on public opinion research				
Low	1,319 (66)	409 (66)	910 (66)	0.996
High	671 (34)	208 (34)	463 (34)	
I trust famous people from social networks				
Low	1,474 (74)	423 (69)	1,051 (77)	< 0.001
High	516 (26)	194 (31)	322 (23)	

The survey results concerning participants’ preventive behaviors and adherence to recommended measures are summarized in Table 3. Approximately 80% or more of respondents reported frequently washing their hands, avoiding touching their eyes, nose, and mouth without washing their hands, using sanitizers, maintaining home isolation, wearing face masks, and disinfecting surfaces. While a high proportion of individuals in both groups adhered to these recommended measures, the percentage of those who used antibiotics without a prescription was consistently higher across all measures, with the differences being statistically significant (Table 3).

**Table 3:** Own behavior for prevention and compliance with prevention measures

Characteristics	Total	Used antibiotics (*n* = 617; 31%)	Didn’t use antibiotics (*n* = 1,373; 69%)	*p*-value
Prevention (own behavior)				
I frequently washed my hands with soap and water for 20 s				
Yes	1,718 (86)	553 (90)	1,165 (85)	0.016
No	239 (12)	57 (9)	182 (13)	
NA	33 (2)	7 (1)	26 (2)	
I avoided touching my eyes, nose and mouth with unwashed hands				
Yes	1,726 (87)	558 (90)	1,168 (85)	0.004
No	222 (11)	48 (8)	174 (13)	
NA	42 (2)	11 (2)	31 (2)	
I used hand sanitizers when there was no soap or water to wash my hands.				
Yes	1,732 (87)	572 (93)	1,160 (85)	< 0.001
No	218 (11)	36 (6)	182 (13)	
NA	40 (2)	9 (1)	31 (2)	
I stayed home and didn’t go to work/school				
Yes	1,652 (83)	546 (88)	1,106 (81)	< 0.001
No	257 (13)	61 (10)	196 (14)	
NA	81 (4)	10 (2)	71 (5)	
I wore a face mask				
Yes	1,862 (93)	594 (96)	1,268 (92)	0.002
No	116 (6)	19 (3)	97 (7)	
NA	12 (1)	4 (1)	8 (1)	
I have maintained physical distancing in public places				
Yes	1,566 (79)	522 (84)	1,044 (76)	< 0.001
No	377 (19)	85 (14)	292 (21)	
NA	47 (2)	10 (2)	37 (3)	
I disinfected surfaces				
Yes	1,550 (78)	536 (87)	1,014 (74)	< 0.001
No	396 (20)	72 (11)	324 (23)	
NA	44 (2)	9 (2)	35 (3)	
Knowledge and self-assessment of compliance with prevention measures				
I am following the recommendations of the authorities in my country to prevent the spread of the new coronavirus				
No	597 (30)	179 (29)	418 (30)	0.519
Yes	1,393 (70)	438 (71)	955 (70)	

The survey findings regarding participants’ worries related to the coronavirus pandemic are detailed in Table 4. While the majority expressed concern about losing loved ones, the proportion of worried individuals was higher among those who did not use antibiotics without a prescription (*p* < 0.001). A similar trend was observed for concerns about the healthcare system becoming overburdened, the country’s economic downturn, the ability to pay bills, and visiting individuals who depend on their care. These differences were statistically significant.Approximately half of the respondents reported concerns about their mental and physical health, restrictions on freedom of movement, the closure of small businesses, limited access to food, and the risk of unemployment (*p* > 0.05).

**Table 4:** Worries related to coronavirus pandemic

Characteristics	Total	Used antibiotics (*n* = 617; 31%)	Didn’t use antibiotics (*n* = 1,373; 69%)	*p*-value
Worry				
How worried are you about losing a loved one?				
Don’t worry	641 (32)	242 (39)	399 (29)	< 0.001
Worry	1,349 (68)	375 (61)	974 (71)	
How concerned are you about the healthcare system becoming overburdened?				
Don’t worry	759 (38)	265 (43)	494 (36)	0.003
Worry	1,231 (62)	352 (57)	879 (64)	
How much do you worry about your mental health?				
Don’t worry	982 (49)	294 (48)	688 (50)	0.310
Worry	1,008 (51)	323 (52)	685 (50)	
How concerned are you about your physical health?				
Don’t worry	870 (44)	277 (45)	593 (43)	0.478
Worry	1,120 (56)	340 (55)	780 (57)	
How much do you worry about the health of your loved ones?				
Don’t worry	626 (31)	250 (41)	376 (27)	< 0.001
Worry	1,364 (69)	367 (59)	997 (73)	
How worried are you about restrictions on freedom of movement?				
Don’t worry	877 (44)	280 (45)	597 (43)	0.430
Worry	1,113 (56)	337 (55)	776 (57)	
How concerned are you about losing your vacation?				
Don’t worry	1,033 (52)	294 (48)	739 (54)	0.011
Worry	957 (48)	323 (52)	634 (46)	
How worried are you about small businesses shutting down?				
Don’t worry	957 (48)	289 (47)	668 (49)	0.454
Worry	1,033 (52)	328 (53)	705 (51)	
How concerned are you about the economic downturn in the country?				
Don’t worry	773 (39)	271 (44)	502 (37)	0.002
Worry	1,217 (61)	346 (56)	871 (63)	
How worried are you about limited access to food?				
Don’t worry	911 (46)	282 (46)	629 (46)	0.965
Worry	1,079 (54)	335 (54)	744 (54)	
How concerned are you about becoming unemployed?				
Don’t worry	890 (45)	293 (47)	597 (43)	0.096
Worry	1,100 (55)	324 (53)	776 (57)	
How worried are you about not being able to pay your bills?				
Don’t worry	814 (41)	278 (45)	536 (39)	0.012
Worry	1,176 (59)	339 (55)	837 (61)	
How concerned are you about not being able to visit people who depend on you?				
Don’t worry	867 (44)	291 (47)	576 (42)	0.030
Worry	1,123 (56)	326 (53)	797 (58)	
How worried are you about having to justify your decision not to attend an event expected by family or friends?				
Don’t worry	1,041 (52)	302 (49)	739 (54)	0.044
Worry	949 (48)	315 (51)	634 (46)	

## Discussion

This study provides a comprehensive analysis of antibiotic use without a prescription among the Kazakhstani population during the COVID-19 pandemic, highlighting significant socio-demographic, behavioral, and attitudinal factors. The findings underscore the complexity of antibiotic misuse and its association with perceptions of the pandemic, preventive behaviors, trust in information sources, and societal concerns.

The higher prevalence of non-prescription antibiotic use among rural residents and those with children under 18 years of age reflects potential disparities in healthcare access and awareness. These results are consistent with prior research from Uganda [10], Tanzania [11], America [12] and China [13] showing that rural populations often have limited access to medical services, leading to increased reliance on self-medication practices. Similarly, caregivers of young children may perceive a greater need for antibiotics to mitigate perceived health risks, particularly during a pandemic. Furthermore, a systematic review on parental self-medication with antibiotics for children found a higher prevalence of this problem in the Middle East (34%) and Asia (20%) [14].

Interestingly, participants who believed they were at high risk of contracting COVID-19 or that the infection could be severe were more likely to use antibiotics without a prescription. This suggests a gap in understanding the nature of viral infections and the ineffectiveness of antibiotics against them. The studies conducted during coronavirus pandemic and before show that people tend to use antibiotics to cure viral infections which lead to higher antimicrobial resistance [15–17]. Public health campaigns should address this misconception, emphasizing the appropriate use of antibiotics to curb antimicrobial resistance.

Regarding trust in information sources, low trust in medical professionals among those who used non-prescription antibiotics is particularly concerning. Medical professionals are critical for guiding appropriate antibiotic use, and diminished trust can undermine efforts to promote rational prescribing. Trust in medical workers and the doctor-patient relationship are critical in public health. Studies examining patient decisions to use non-prescribed antibiotics have revealed that individuals are less likely to engage in such practices if they trust general practitioners and if the consequences of reckless antibiotic use, including its contribution to AMR and its severe complications, are clearly explained [18–20]. The high levels of distrust in traditional and social media as reliable information sources further complicate public health communication strategies. These findings align with global studies indicating that misinformation and distrust in healthcare systems exacerbate antibiotic misuse during health crises [21]. Targeted communication strategies that enhance trust in healthcare providers and deliver clear, evidence-based messages are urgently needed.

A global survey during COVID-19 pandemic conducted across 28 countries found that approximately one-third of respondents believed that “a foreign power or other force” intentionally caused the pandemic. For instance, this belief was held by 18% of respondents in the United Kingdom, 58% in Bulgaria, and 26% in Thailand [22]. Such conspiracy theories pose a significant public health challenge, as they erode trust in government authorities and healthcare professionals - a pattern also observed during previous disease outbreaks [23]. In this study, an unexpected yet intriguing finding was the lower prevalence of non-prescription antibiotic use among participants who endorsed conspiracy theories about hidden motives behind global events. This could indicate that individuals skeptical of mainstream narratives might also reject conventional healthcare practices, including antibiotic use. Research has shown that conspiracy beliefs are often linked to a preference for alternative remedies [24], which could explain this trend. Future studies should explore whether these individuals are replacing antibiotics with other treatments or avoiding medical interventions altogether.

Participants of this study commonly engaged in preventive measures such as regular handwashing, wearing masks, and disinfecting surfaces. A similar pattern was observed in a global study examining shifts in adherence to COVID-19 protective behaviors. The findings indicated that low-cost, habitual practices like mask-wearing showed a steady increase in adherence, while high-effort measures such as physical distancing experienced a decline over pandemic period [25]. However, the higher prevalence of non-prescription antibiotic use among those adhering to these measures in this study suggests that compliance with public health recommendations does not necessarily translate into appropriate antibiotic use. In addition, participants’ worries about the pandemic, including concerns about losing loved ones and systemic issues such as economic downturns and overburdened healthcare systems, were significantly associated with antibiotic use patterns. In addition, online misinformation could promote self-medication, as public distrust in hospitals could drive reliance on unverified sources, increasing health risks during the pandemic [26]. These findings suggest that emotional and societal stressors during health crises may drive self-medication practices, underscoring the need for psychosocial support and public health interventions that address broader societal concerns alongside medical education.

This study contributes directly to Sustainable Development Goals (SDG) 3 – health and well-being – and 10 – reduction of inequality – by addressing the critical issue of non-prescription antibiotic use, a public health challenge with significant implications for AMR. By identifying socio-demographic disparities, trust dynamics, and behavioral drivers of antibiotic misuse, this research provides actionable insights that can inform national strategies to promote antibiotic stewardship and enhance healthcare equity. Such efforts align with the SDG 3 target of ensuring access to safe and effective medicines while combating AMR, a growing global concern. Furthermore, the study emphasizes the importance of health literacy, trust in healthcare systems, and tailored public health interventions, which support Kazakhstan’s ongoing progress toward achieving SDG-related health objectives. Strengthening communication campaigns that rebuild trust in healthcare providers, alongside educational initiatives that improve health literacy, can help reduce reliance on non-prescription antibiotics. Additionally, regulatory measures restricting over-the-counter antibiotic sales are essential to curb self-medication practices.

This study has several strengths and limitations. Among its strengths, the research addresses a critical public health issue – non-prescription antibiotic use – during the COVID-19 pandemic, a period marked by increased self-medication practices. The study’s broad scope, encompassing socio-demographic factors, perceptions, behaviors, trust in information sources, and conspiracy beliefs, provides a comprehensive understanding of the drivers of antibiotic misuse. The adaptation of the WHO Behavioral Insights Tool to the local context of Kazakhstan ensures cultural and linguistic relevance, enhancing the reliability of the findings. However, the study’s cross-sectional design limits the ability to establish causality, and the snowball sampling method may introduce selection bias. The reliance on self-reported data is subject to recall and social desirability bias. Furthermore, while the study provides valuable insights into Kazakhstan’s context, the findings may not be directly generalizable to other countries or regions with differing healthcare systems and cultural practices.

## Conclusion

The findings of this study reveal disparities in healthcare access, particularly among rural residents and caregivers of young children, and emphasize the critical role of trust in medical professionals in mitigating antibiotic misuse. The study also identifies a concerning association between low trust in healthcare providers and higher rates of self-medication, alongside a nuanced relationship between conspiracy beliefs and antibiotic use. These insights underscore the importance of improving health literacy and strengthening public health initiatives to enhance awareness about the risks of antibiotic misuse. Additionally, they highlight the need for governmental efforts to address healthcare inequalities, build trust in medical systems, and promote evidence-based practices, ultimately supporting effective antibiotic stewardship.

## Supplementary Information

Supplementary Material 1.

## Data Availability

The data that support the findings of this study are available from the Ministry of Health of the republic of Kazakhstan, but restrictions apply to the availability of these data, which were used under the contract agreement for the current study, and so are not publicly available. Data are however available from the corresponding author, N.A., upon reasonable request and with permission of the Ministry of Health of the Republic of Kazakhstan.

## Abbreviations

AMR: Antimicrobial resistance
COSMO: COVID-19 Snapshot Monitoring
GDP: gross domestic product
WHO: World Health Organization
