# Supplementary material for: Understanding antibiotic misuse in Kazakhstan: insights from the WHO behavioral survey on COVID-19
Source: BMC Infect Dis. 2025 Oct 16;25:1341. doi: 10.1186/s12879-025-11764-y (PMC12532856; doi:10.1186/s12879-025-11764-y)
Supplement: Supplementary file 1 — Supplementary Material 1. [file 12879_2025_11764_MOESM1_ESM.docx]

Questionnaire

1. How old are you?
2. What’s your gender?
3. Female
4. Male
5. Where do you live?
6. Urban area
7. Rural area
8. Are you a medical worker?
9. Yes
10. No
11. Do you have children under 18 years of age living with you?
12. Yes
13. No
14. Are there people over 60 and/or with chronic illnesses living with you?
15. Yes
16. No

Probability and severity of COVID-19 (perception)

1. How likely do you think you will become infected with COVID-19?
2. Very unlikely
3. Unlikely
4. Neutral
5. Likely
6. Very likely
7. How severe could a COVID-19 infection be for you?
   1. Not severe at all
   2. Mildly severe
   3. Moderately severe
   4. Quite severe
   5. Extremely severe

Readiness and perception of own capabilities

1. I know how to protect myself from coronavirus
2. Don’t know at all
3. Know a little
4. Partially know
5. Know well
6. Know completely
7. For me, avoiding contracting COVID-19 in the current situation is
8. Very difficult
9. Difficult
10. Neutral
11. Easy
12. Very easy

Prevention (own behavior)

Recently, which of the following measures have you taken to prevent infection with COVID-19?

1. I frequently washed my hands with soap and water for 20 seconds
2. Not at all
3. Rarely
4. Sometimes
5. Often
6. Very often
7. Not applicable
8. I avoided touching my eyes, nose, and mouth with unwashed hands
9. Not at all
10. Rarely
11. Sometimes
12. Often
13. Very often
14. Not applicable
15. I used hand sanitizers when there was no soap or water to wash my hands.
16. Not at all
17. Rarely
18. Sometimes
19. Often
20. Very often
21. Not applicable
22. I stayed home and didn't go to work/school
23. Not at all
24. Rarely
25. Sometimes
26. Often
27. Very often
28. Not applicable
29. I wore a face mask
30. Not at all
31. Rarely
32. Sometimes
33. Often
34. Very often
35. Not applicable
36. I have maintained physical distancing in public places (at least 1.5 meters between you and other people outside your home)
37. Not at all
38. Rarely
39. Sometimes
40. Often
41. Very often
42. Not applicable
43. I disinfected surfaces
44. Not at all
45. Rarely
46. Sometimes
47. Often
48. Very often
49. Not applicable

Knowledge and self-assessment of compliance with prevention measures

1. I am following the recommendations of the authorities in my country to prevent the spread of the new coronavirus
2. Do not follow at all
3. Follow a little
4. Follow some recommendations
5. Follow most recommendations
6. Follow all recommendations

Trust in information sources

How much do you trust the following information sources for their reporting on the novel coronavirus?

1. I trust television
2. Very low level of trust
3. Low level of trust
4. Moderate level of trust
5. High level of trust
6. Very high level of trust
7. I trust daily or weekly newspapers
8. Very low level of trust
9. Low level of trust
10. Moderate level of trust
11. High level of trust
12. Very high level of trust
13. I trust conversations with family, friends, colleagues
14. Very low level of trust
15. Low level of trust
16. Moderate level of trust
17. High level of trust
18. Very high level of trust
19. I trust consultations with medical professionals
20. Very low level of trust
21. Low level of trust
22. Moderate level of trust
23. High level of trust
24. Very high level of trust
25. I trust social networks (for example, Facebook, Twitter, YouTube, Telegram, WhatsApp, Instagram)
26. Very low level of trust
27. Low level of trust
28. Moderate level of trust
29. High level of trust
30. Very high level of trust
31. I trust the radio station
32. Very low level of trust
33. Low level of trust
34. Moderate level of trust
35. High level of trust
36. Very high level of trust
37. I rely on public opinion research
38. Very low level of trust
39. Low level of trust
40. Moderate level of trust
41. High level of trust
42. Very high level of trust
43. I trust famous people from social networks
44. Very low level of trust
45. Low level of trust
46. Moderate level of trust
47. High level of trust
48. Very high level of trust

Conspiracy Theories (Perception)

Please rate your agreement or disagreement with the following statements on a 5-point scale.

1. There are many very important things happening in the world that the public is never informed about.
2. Definitely false
3. Mostly false
4. Uncertain
5. Mostly true
6. Definitely true
7. Politicians usually don't tell us the true reasons behind their decisions
8. Definitely false
9. Mostly false
10. Uncertain
11. Mostly true
12. Definitely true
13. Government agencies closely monitor all citizens
14. Definitely false
15. Mostly false
16. Uncertain
17. Mostly true
18. Definitely true
19. Events that seem unrelated are often the result of covert activity
20. Definitely false
21. Mostly false
22. Uncertain
23. Mostly true
24. Definitely true
25. There are secret organizations that have a very strong influence on political decisions.
26. Definitely false
27. Mostly false
28. Uncertain
29. Mostly true
30. Definitely true

Worry

1. How worried are you about losing a loved one?
2. Not worried at all
3. Slightly worried
4. Moderately worried
5. Quite worried
6. Very worried
7. How concerned are you about the healthcare system becoming overburdened?
8. Not worried at all
9. Slightly worried
10. Moderately worried
11. Quite worried
12. Very worried
13. How much do you worry about your mental health?
14. Not worried at all
15. Slightly worried
16. Moderately worried
17. Quite worried
18. Very worried
19. How concerned are you about your physical health?
20. Not worried at all
21. Slightly worried
22. Moderately worried
23. Quite worried
24. Very worried
25. How much do you worry about the health of your loved ones?
26. Not worried at all
27. Slightly worried
28. Moderately worried
29. Quite worried
30. Very worried
31. How worried are you about restrictions on freedom of movement?
32. Not worried at all
33. Slightly worried
34. Moderately worried
35. Quite worried
36. Very worried
37. How concerned are you about losing your vacation?
38. Not worried at all
39. Slightly worried
40. Moderately worried
41. Quite worried
42. Very worried
43. How worried are you about small businesses shutting down?
44. Not worried at all
45. Slightly worried
46. Moderately worried
47. Quite worried
48. Very worried
49. How concerned are you about the economic downturn in the country?
50. Not worried at all
51. Slightly worried
52. Moderately worried
53. Quite worried
54. Very worried
55. How worried are you about limited access to food?
56. Not worried at all
57. Slightly worried
58. Moderately worried
59. Quite worried
60. Very worried
61. How concerned are you about becoming unemployed?
62. Not worried at all
63. Slightly worried
64. Moderately worried
65. Quite worried
66. Very worried
67. How worried are you about not being able to pay your bills?
68. Not worried at all
69. Slightly worried
70. Moderately worried
71. Quite worried
72. Very worried
73. How concerned are you about not being able to visit people who depend on you?
74. Not worried at all
75. Slightly worried
76. Moderately worried
77. Quite worried
78. Very worried
79. How worried are you about having to justify your decision not to attend an event expected by family or friends?
80. Not worried at all
81. Slightly worried
82. Moderately worried
83. Quite worried
84. Very worried
